# Cattaneo-Christov Heat Flux Model for MHD Three-Dimensional Flow of Maxwell Fluid over a Stretching Sheet

**DOI:** 10.1371/journal.pone.0153481

**Published:** 2016-04-19

**Authors:** Khansa Rubab, M. Mustafa

**Affiliations:** School of Natural Sciences (SNS), National University of Sciences and Technology (NUST), Islamabad 44000, Pakistan; Universidad Rey Juan Carlos, SPAIN

## Abstract

This letter investigates the MHD three-dimensional flow of upper-convected Maxwell (UCM) fluid over a bi-directional stretching surface by considering the Cattaneo-Christov heat flux model. This model has tendency to capture the characteristics of thermal relaxation time. The governing partial differential equations even after employing the boundary layer approximations are non linear. Accurate analytic solutions for velocity and temperature distributions are computed through well-known homotopy analysis method (HAM). It is noticed that velocity decreases and temperature rises when stronger magnetic field strength is accounted. Penetration depth of temperature is a decreasing function of thermal relaxation time. The analysis for classical Fourier heat conduction law can be obtained as a special case of the present work. To our knowledge, the Cattaneo-Christov heat flux model law for three-dimensional viscoelastic flow problem is just introduced here.

## Introduction

The phenomenon of heat transfer has widespread industrial and biomedical applications such as cooling of electronic devices, nuclear reactor cooling, power generation, heat conduction in tissues and many others. The heat flux model proposed by Fourier [1] has been the most successful model for understanding heat transfer mechanism in diverse situations. One of the limitations of this model is that it often leads to a parabolic energy equation which indicates that initial disturbance is instantly experienced by the medium under consideration. This physically unrealistic feature is referred in the literature as “Paradox of heat conduction”. In order to overcome this enigma, various researchers have proposed alterations in the Fourier’s heat conduction law. Cattaneo [2] modified this law through the inclusion of thermal relaxation time which is defined as the time required establishing heat conduction once the temperature gradient is imposed. Christov [3] further modified the Cattaneo model by replacing the ordinary derivative with the Oldroyd’s upper-convected derivative. He also presented the energy equation for arbitrary velocity and temperature fields. Straughan [4] applied Cattaneo-Christov model to study thermal convection in horizontal layer of incompressible Newtonian fluid under the influence of gravity. Ciarletta and Straughan [5] proved the uniqueness and stability of the solutions for the Cattaneo-Christov equations. Tibullo and Zampoli [6] investigated the uniqueness of solutions for an incompressible flow problem by using Cattaneo-Christov model. Han et al. [7] considered the two-dimensional flow and heat transfer of viscoelastic fluid over a stretching sheet using the Cattaneo-Christov heat flux model. In this study the analytic solutions were achieved by homotopy analysis method (HAM). Mustafa [8] developed both numerical and homotopy solutions for rotating flow of Maxwell fluid through Cattaneo-Christov theory. Later, Khan et al. [9] presented numerical approximations for viscoelastic flow over an exponentially stretching surface with the consideration of Cattaneo-Christov model. In a recent paper Hayat et al. [10] discussed the impact of Cattaneo-Christov heat conduction on the flow problem involving oldroyd-B fluid.

The analysis of magnetohydrodynamic (MHD) in viscous or non-newtonian flow is important in MHD generators, plasma studies, thermal therapy for cancer treatment, contrast enhancement in magnetic resonance imaging (MRI), nuclear reactors, geothermal energy extraction and many others. More precisely, MHD flow caused by the deformation of the walls of vessel containing the fluid has special value in modern metallurgical and metal working processes. Several recent attempts have been put forward in this direction in which Zheng et al. [11] studied the velocity slip and temperature jump conditions for MHD flow and heat transfer due to shrinking surface. Gul et al. [12] used Adomian Decomposition Method (ADM) to investigate the thin film flow of third grade fluid under the influence of magnetic field. In another paper, Gul et al. [13] analytically explored the heat transfer characteristic for unsteady MHD thin film flow of second grade fluid using two different approaches. Unsteady MHD thin film flow of Oldroyd-B fluid was discussed by Gul et al. [14]. Mixed convection flow of nanofluid under the influence of magnetic force was numerically explored by Dhanai et al. [15]. Mabood et al. [16] describe the influence of magnetic field on the nanofluid flow driven by a non-linearly stretching surface. Second order slip effects on the boundary layer flow of nanofluid adjacent to stretching/shrinking sheet were discussed by Abdul Hakeem et al. [17]. Rashidi et al. [18] numerically explored the magnetic field effects on mixed convection flow of nanofluid in a vertical channel having sinusoidal walls. Hayat et al. [19] analytically investigates the peristaltic transport of in inclined channel under inclined magnetic field effects. In another paper Hayat et al. [20] discussed the MHD peristaltic motion of nanofluid in complaint wall channel.

Present work is undertaken to study the heat transfer in MHD three-dimensional flow of upper-convected Maxwell fluid by using Cattaneo-Christov heat flux model. Maxwell fluid is one of the popular viscoelastic models that can address the influence of fluid relaxation time. The boundary layer flows of Maxwell fluid have received remarkable attention in the past. Some interesting flow problems involving Maxwell fluid can be found in refs. [21–30]. The equations are formulated and then solved for convergence of series solution by homotopic approach. Liao [31] proposed the homotopy analysis method (HAM) which is based on homotopy, a fundamental concept of topology and differential geometry. This method is considered to be better than other approximate analytical methods due to various reasons. For instance, perturbation techniques require the existence of perturbation quantity in the problem. However most of the non-linear problems in science and engineering do not contain such quantities. This serious restriction makes the perturbation methods valid only for weakly non-linear problems. Unlike non-perturbation approaches namely homotopy perturbation method (HPM), Adomian Decomposition method and *δ*-expansion method, HAM provides flexibility to choose proper base functions in order to get better approximation of the solutions. Moreover HAM provides a convenient way to control the convergence of series solutions in the form of an auxiliary parameter ℏ [32]. Graphs are sketched to see the influence of important parameters on the velocity and temperature fields.

## Problem formulation

**Fig 1:**
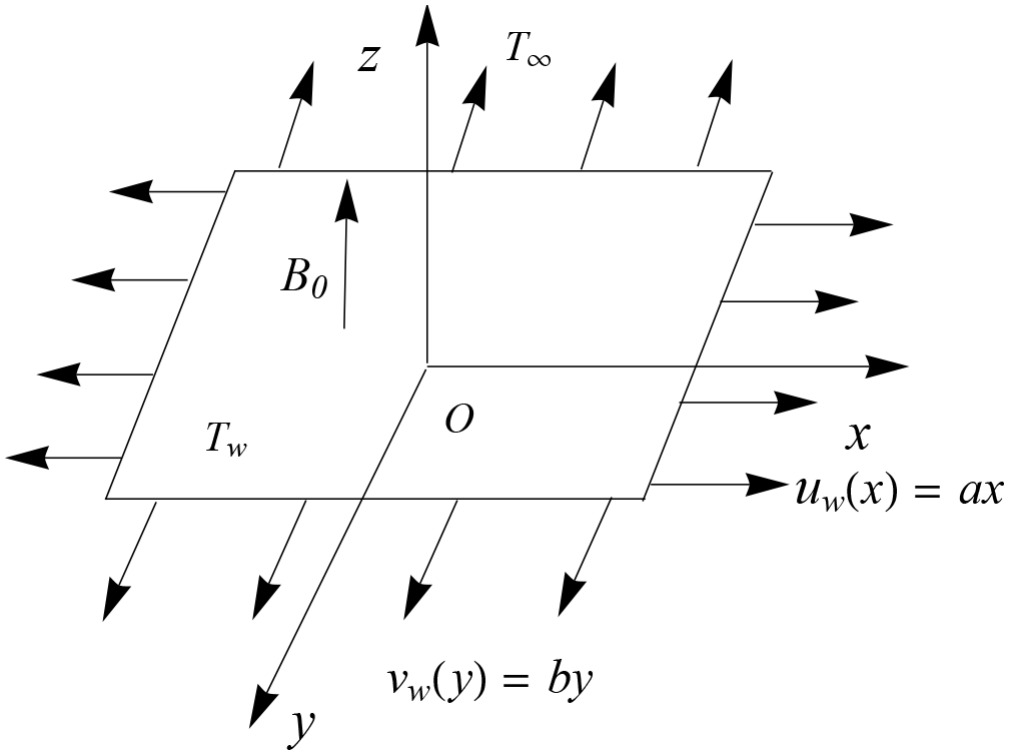
Geometry of the problem.

Consider the flow of upper-convected Maxwell fluid induced by an elastic sheet stretching in two lateral directions. The sheet is coincident with the plane *z* = 0, whereas the fluid occupies the region *z* ≥ 0. The electric field is absent while induced magnetic field is neglected due to the consideration of small magnetic Reynolds number. The velocities of the stretching sheet along the *x*− and *y*− directions are *u*_*w*_(*x*) = *ax* and *v*_*w*_(*y*) = *by* respectively. The sheet is kept at constant temperature *T*_*w*_, whereas *T*_∞_ is the ambient value of the temperature such that *T*_*w*_ > *T*_∞_. Considering the velocity vector *V* = [*u*(*x*, *y*, *z*), *v*(*x*, *y*, *z*), *w*(*x*, *y*, *z*)] and the temperature *T* (see Fig 1). The boundary layer equations for three-dimensional flow and heat transfer of Maxwell fluid can be expressed as below:

$$\begin{array}{ccc} \frac{\partial u}{\partial x}+\frac{\partial v}{\partial y}+\frac{\partial w}{\partial z} & = & 0, \end{array}$$ (1)

$$\begin{array}{ccc} u\frac{\partial u}{\partial x}+v\frac{\partial u}{\partial y}+w\frac{\partial u}{\partial z} & = & \nu \frac{\partial^{2}u}{\partial z^{2}}-\lambda_{1}(u^{2}\frac{\partial^{2}u}{\partial x^{2}}+v^{2}\frac{\partial^{2}u}{\partial y^{2}}+w^{2}\frac{\partial^{2}u}{\partial z^{2}}+2uv\frac{\partial^{2}u}{\partial x\partial y}\\ & & +2vw\frac{\partial^{2}u}{\partial y\partial z}+2uw\frac{\partial^{2}u}{\partial x\partial z})-\frac{\sigma B_{0}^{2}}{\rho }(u+\lambda_{1}w\frac{\partial u}{\partial z}), \end{array}$$ (2)

$$\begin{array}{ccc} u\frac{\partial v}{\partial x}+v\frac{\partial v}{\partial y}+w\frac{\partial v}{\partial z} & = & \nu \frac{\partial^{2}v}{\partial z^{2}}-\lambda_{1}(u^{2}\frac{\partial^{2}v}{\partial x^{2}}+v^{2}\frac{\partial^{2}v}{\partial y^{2}}+w^{2}\frac{\partial^{2}v}{\partial z^{2}}+2uv\frac{\partial^{2}v}{\partial x\partial y}\\ & & +2vw\frac{\partial^{2}v}{\partial y\partial z}+2uw\frac{\partial^{2}v}{\partial x\partial z})-\frac{\sigma B_{0}^{2}}{\rho }(v+\lambda_{1}w\frac{\partial v}{\partial z}), \end{array}$$ (3)

$$\begin{array}{ccc} \rho c_{p}(u\frac{\partial T}{\partial x}+v\frac{\partial T}{\partial y}+w\frac{\partial T}{\partial z}) & = & -\nabla .q, \end{array}$$ (4)

where *u*, *v* and *w* are the velocity components along the *x*−, *y*− and *z*− directions respectively, *ν* is the kinematic viscosity, *c*_*p*_ is the specific heat, *σ* is the electrical conductivity, *ρ* is the fluid density, *T* is the fluid temperature, λ_1_ is the fluid relaxation time and **q** is the heat flux which satisfies the following relationship [3].

$$\begin{array}{c} q+\lambda_{2}\left[\frac{\partial q}{\partial t}+V.\nabla q-q.\nabla V+\left(\nabla .V\right)q\right]=-k\nabla T, \end{array}$$ (5)

in which λ_2_ is the thermal relaxation time and *k* is the thermal conductivity of the fluid. Following Christov [3], we eliminate **q** from Eqs (4) and (5) to obtain the following:

$$\begin{array}{ccc} u\frac{\partial T}{\partial x}+v\frac{\partial T}{\partial y}+w\frac{\partial T}{\partial z} & = & \frac{k}{\rho c_{p}}\frac{\partial^{2}T}{\partial z^{2}}-\lambda_{2}[u^{2}\frac{\partial^{2}T}{\partial x^{2}}+v^{2}\frac{\partial^{2}T}{\partial y^{2}}+w^{2}\frac{\partial^{2}T}{\partial z^{2}}+2uv\frac{\partial^{2}T}{\partial x\partial y}+\\ & & 2vw\frac{\partial^{2}T}{\partial y\partial z}+2uw\frac{\partial^{2}T}{\partial x\partial z}+\left(u\frac{\partial u}{\partial x}+v\frac{\partial u}{\partial y}+w\frac{\partial u}{\partial z}\right)\frac{\partial T}{\partial x}+(u\\ & & \frac{\partial v}{\partial x}+v\frac{\partial v}{\partial y}+w\frac{\partial v}{\partial z})\frac{\partial T}{\partial y}+\left(u\frac{\partial w}{\partial x}+v\frac{\partial w}{\partial y}+w\frac{\partial w}{\partial z}\right)\frac{\partial T}{\partial z}] \end{array}$$ (6)

Boundary conditions for the present problem are:

$$\begin{array}{c} u=u_{w}\left(x\right)=ax,\;v=v_{w}\left(y\right)=by,\;w=0,\;T=T_{w}\text{ at }z=0,\\ u\to 0,\;v\to 0,\;T\to T_{\infty }\;as\;z\to \infty . \end{array}$$ (7)

Considering the following similarity transformations

$$\begin{array}{c} \begin{array}{cc} & \;\eta =\sqrt{\frac{a}{\nu }}z,\;\;u=axf^{\prime }\left(\eta \right),\;\;v=ayg^{\prime }\left(\eta \right),\;\;w=-\sqrt{a\nu }\left[f\left(\eta \right)+g\left(\eta \right)\right],\\ & \;\theta =\frac{T-T_{\infty }}{T_{w}-T_{\infty }}. \end{array} \end{array}$$ (8)

Eq (1) is identically satisfied and Eqs (2), (3), (4) and (5) take the following forms:

$$\begin{array}{c} f^{\prime \prime \prime }+\left(M^{2}\beta +1\right)\left(f+g\right)f^{\prime \prime }-f^{\prime 2}+\beta \left[2\left(f+g\right)f^{\prime }f^{\prime \prime }-{\left(f+g\right)}^{2}f^{\prime \prime \prime }\right]-M^{2}f^{\prime }=0, \end{array}$$ (9)

$$\begin{array}{c} g^{\prime \prime \prime }+\left(M^{2}\beta +1\right)\left(f+g\right)g^{\prime \prime }-g^{\prime 2}+\beta \left[2\left(f+g\right)g^{\prime }g^{\prime \prime }-{\left(f+g\right)}^{2}g^{\prime \prime \prime }\right]-M^{2}g^{\prime }=0, \end{array}$$ (10)

$$\begin{array}{c} \frac{1}{Pr}\theta^{\prime \prime }+\left(f+g\right)\theta^{\prime }-\gamma \left[\left(f+g\right)\left(f^{\prime }+g^{\prime }\right)\theta^{\prime }+{\left(f+g\right)}^{2}\theta^{\prime \prime })\right]=0, \end{array}$$ (11)

$$\begin{array}{c} \begin{array}{cc} & \;f\left(0\right)=g\left(0\right)=0,\;\;f^{\prime }\left(0\right)=1,\;\;g^{\prime }\left(0\right)=\lambda ,\;\;\theta \left(0\right)=1,\\ & \;f^{\prime }\left(\infty \right)\to 0,\;\;g^{\prime }\left(\infty \right)\to 0,\;\;\theta \left(\infty \right)\to 0, \end{array} \end{array}$$ (12)

where λ = *b*/*a* is the ratio of the stretching rate along the *y*− direction to the stretching rate along the *x*− direction, *β* = λ_1_ *a* is the non-dimensional fluid relaxation time, *γ* = λ_2_ *a* is the non-dimensional relaxation time for heat flux and *Pr* is the Prandtl number. It can be noticed that when λ = 0, the two-dimensional case is jumped. Further λ = 1 corresponds to the case of axisymmetric flow.

## Analytic solutions by homotopy analysis method

In this section, we deal with series solutions by homotopy analysis method (HAM) [31] for non-linear coupled eqs (9), (10) and (11) with boundary conditions Eq (12). In order to proceed, we choose initial approximations for functions *f*_0_, *g*_0_ and *θ*_0_ as follows:

$$\begin{array}{c} f_{0}\left(\eta \right)=1-e^{-\eta },\;\;\;g_{0}\left(\eta \right)=\lambda \left(1-e^{-\eta }\right),\;\;\;\theta_{0}\left(\eta \right)=e^{-\eta }. \end{array}$$ (13)

The auxiliary linear operators $L_{f}$, $L_{g}$ and $L_{\theta }$ are selected as

$$\begin{array}{c} L_{f}\left(\eta \right)=f^{\prime \prime \prime }-f^{\prime },\;\;\;L_{g}\left(\eta \right)=g^{\prime \prime \prime }-g^{\prime },\;\;\;L_{\theta }\left(\eta \right)=\theta^{\prime \prime }-\theta . \end{array}$$ (14)

Now consider the non-linear operators $N_{f}$, $N_{g}$ and $N_{\theta }$ as below:

$$\begin{array}{ccc} N_{f}\left[\hat{f}\left(\eta ;p\right),\hat{g}\left(\eta ;p\right)\right] & = & \frac{\partial^{3}\hat{f}\left(\eta ;p\right)}{\partial \eta^{3}}-{\left(\frac{\partial \hat{f}\left(\eta ;p\right)}{\partial \eta }\right)}^{2}+\left(M^{2}\beta +1\right)\left(\hat{f}\left(\eta ;p\right)+\hat{g}\left(\eta ;p\right)\right)\\ & & \;\frac{\partial^{2}\hat{f}\left(\eta ;p\right)}{\partial \eta^{2}}+\beta (2\left(\hat{f}\left(\eta ;p\right)+\hat{g}\left(\eta ;p\right)\right)\frac{\partial \hat{f}\left(\eta ;p\right)}{\partial \eta }\frac{\partial^{2}\hat{f}\left(\eta ;p\right)}{\partial \eta^{2}}-\\ & & {\left(\hat{f}\left(\eta ;p\right)+\hat{g}\left(\eta ;p\right)\right)}^{2}\frac{\partial^{3}\hat{f}\left(\eta ;p\right)}{\partial \eta^{3}})-M^{2}\frac{\partial \hat{f}\left(\eta ;p\right)}{\partial \eta }, \end{array}$$ (15)

$$\begin{array}{ccc} N_{g}\left[\hat{g}\left(\eta ;p\right),\hat{f}\left(\eta ;p\right)\right] & = & \frac{\partial^{3}\hat{g}\left(\eta ;p\right)}{\partial \eta^{3}}-{\left(\frac{\partial \hat{g}\left(\eta ;p\right)}{\partial \eta }\right)}^{2}+\left(M^{2}\beta +1\right)\left(\hat{f}\left(\eta ;p\right)+\hat{g}\left(\eta ;p\right)\right)\\ & & \frac{\partial^{2}\hat{g}\left(\eta ;p\right)}{\partial \eta^{2}}+\beta (2\left(\hat{f}\left(\eta ;p\right)+\hat{g}\left(\eta ;p\right)\right)\frac{\partial \hat{g}\left(\eta ;p\right)}{\partial \eta }\frac{\partial^{2}\hat{g}\left(\eta ;p\right)}{\partial \eta^{2}}\\ & & -{\left(\hat{f}\left(\eta ;p\right)+\hat{g}\left(\eta ;p\right)\right)}^{2}\frac{\partial^{3}\hat{g}\left(\eta ;p\right)}{\partial \eta^{3}})-M^{2}\frac{\partial \hat{g}\left(\eta ;p\right)}{\partial \eta }, \end{array}$$ (16)

$$\begin{array}{ccc} N_{\theta }\left[\hat{\theta }\left(\eta ;p\right),\hat{f}\left(\eta ;p\right),\hat{g}\left(\eta ;p\right)\right] & = & \frac{1}{Pr}\frac{\partial^{2}\hat{\theta }\left(\eta ;p\right)}{\partial \eta^{2}}+\left(\hat{f}\left(\eta ;p\right)+\hat{g}\left(\eta ;p\right)\right)\frac{\partial \hat{\theta }\left(\eta ;p\right)}{\partial \eta }-\gamma ((\hat{f}\left(\eta ;p\right)\\ & & +\hat{g}\left(\eta ;p\right))\left(\frac{\partial \hat{f}\left(\eta ;p\right)}{\partial \eta }+\frac{\partial \hat{g}\left(\eta ;p\right)}{\partial \eta }\right)\frac{\partial \hat{\theta }\left(\eta ;p\right)}{\partial \eta }+{\left(\hat{f}\left(\eta ;p\right)+\hat{g}\left(\eta ;p\right)\right)}^{2}\frac{\partial^{2}\hat{\theta }\left(\eta ;p\right)}{\partial \eta^{2}}). \end{array}$$ (17)

The auxiliary linear operators in Eq (14) satisfy the following:

$$\begin{array}{c} L_{f}\left(C_{1}+C_{2}e^{\eta }+C_{3}e^{-\eta }\right)=0,\;\;\;L_{g}\left(C_{4}+C_{5}e^{\eta }+C_{6}e^{-\eta }\right)=0,\;\;\;L_{\theta }\left(C_{7}e^{\eta }+C_{8}e^{-\eta }\right)=0, \end{array}$$ (18)

in which *C*_*i*_(*i* = 1−8) are constants.

Following the basic idea of HAM [31], we express the zeroth-order deformation problems for Eqs (9)–(11) are listed as

$$\begin{array}{c} \left(1-p\right)L_{f}\left[\hat{f}\left(\eta ;p\right)-f_{0}\left(\eta \right)\right]=p\hbar N_{f}\left[\hat{f}\left(\eta ;p\right),\hat{g}\left(\eta ;p\right)\right], \end{array}$$ (19)

$$\begin{array}{c} \left(1-p\right)L_{g}\left[\hat{g}\left(\eta ;p\right)-g_{0}\left(\eta \right)\right]=p\hbar N_{g}\left[\hat{f}\left(\eta ;p\right),\hat{g}\left(\eta ;p\right)\right], \end{array}$$ (20)

$$\begin{array}{c} \left(1-p\right)L_{\theta }\left[\hat{\theta }\left(\eta ;p\right)-\theta_{0}\left(\eta \right)\right]=p\hbar N_{\theta }\left[\hat{f}\left(\eta ;p\right),\hat{g}\left(\eta ;p\right),\hat{\theta }\left(\eta ;p\right)\right]. \end{array}$$ (21)

The boundary-conditions are

$$\begin{array}{ll} \hat{f}\left(\eta ;p\right){|}_{\eta =0}= & 0,\;\frac{\partial \hat{f}\left(\eta ;p\right)}{\partial \eta }{|}_{\eta =0}=1,\;\frac{\partial \hat{f}\left(\eta ;p\right)}{\partial \eta }{|}_{\eta \to \infty }=0,\\ \hat{g}\left(\eta ;p\right){|}_{\eta =0}= & 0,\;\frac{\partial \hat{g}\left(\eta ;p\right)}{\partial \eta }{|}_{\eta =0}=\lambda ,\;\frac{\partial \hat{g}\left(\eta ;p\right)}{\partial \eta }{|}_{\eta \to \infty }=0,\\ \hat{\theta }\left(\eta ;p\right){|}_{\eta =0}= & 1,\;\hat{\theta }\left(\eta ;p\right){|}_{\eta \to \infty }=0, \end{array}$$ (22)

where *p* ∈ [0, 1] is an embedding parameter and ℏ is the non-zero convergence control parameter. When *p* = 0 and *p* = 1 we have:

$$\begin{array}{c} \hat{f}\left(\eta ;0\right)=f_{0}\left(\eta \right),\;\;\;\hat{g}\left(\eta ;0\right)=g_{0}\left(\eta \right),\;\;\;\hat{\theta }\left(\eta ;0\right)=\theta_{0}\left(\eta \right),\\ \hat{f}\left(\eta ;1\right)=f\left(\eta \right),\;\;\;\hat{g}\left(\eta ;1\right)=g\left(\eta \right),\;\;\;\hat{\theta }\left(\eta ;1\right)=\theta \left(\eta \right). \end{array}$$ (23)

Now expanding $\hat{f}\left(\eta ;p\right)$, $\hat{g}\left(\eta ;p\right)$ and $\hat{\theta }\left(\eta ;p\right)$ in Taylor’s series about *p* = 0.

$$\begin{array}{c} \hat{f}\left(\eta ;p\right)=f_{0}\left(\eta \right)+\sum_{m=1}^{\infty }f_{m}\left(\eta \right)p^{m}, \end{array}$$ (24)

$$\begin{array}{c} \hat{g}\left(\eta ;p\right)=g_{0}\left(\eta \right)+\sum_{m=1}^{\infty }g_{m}\left(\eta \right)p^{m}, \end{array}$$ (25)

$$\begin{array}{c} \hat{\theta }\left(\eta ;p\right)=\theta_{0}\left(\eta \right)+\sum_{m=1}^{\infty }\theta_{m}\left(\eta \right)p^{m}, \end{array}$$ (26)

$$\begin{array}{ll} \hat{f}\left(\eta ;p\right){|}_{\eta =0}= & 0,\;\frac{\partial \hat{f}\left(\eta ;p\right)}{\partial \eta }{|}_{\eta =0}=1,\;\frac{\partial \hat{f}\left(\eta ;p\right)}{\partial \eta }{|}_{\eta \to \infty }=0,\\ \hat{g}\left(\eta ;p\right){|}_{\eta =0}= & 0,\;\frac{\partial \hat{g}\left(\eta ;p\right)}{\partial \eta }{|}_{\eta =0}=\lambda ,\;\frac{\partial \hat{g}\left(\eta ;p\right)}{\partial \eta }{|}_{\eta \to \infty }=0,\\ \hat{\theta }\left(\eta ;p\right){|}_{\eta =0}= & 1,\;\hat{\theta }\left(\eta ;p\right){|}_{\eta \to \infty }=0. \end{array}$$ (27)

where

$$\begin{array}{c} f_{m}\left(\eta \right)=\frac{1}{m!}\frac{\partial^{m}\hat{f}\left(\eta ;p\right)}{\partial p^{m}}{|}_{p=0},\;\;g_{m}\left(\eta \right)=\frac{1}{m!}\frac{\partial^{m}\hat{g}\left(\eta ;p\right)}{\partial p^{m}}{|}_{p=0},\;\;\theta_{m}\left(\eta \right)=\frac{1}{m!}\frac{\partial^{m}\hat{\theta }\left(\eta ;p\right)}{\partial p^{m}}{|}_{p=0}. \end{array}$$

The auxiliary parameter ℏ can be chosen in such a way that the series Eqs (24)–(26) converges at *p* = 1. Substituting *p* = 1 in Eqs (24)–(26), we obtain

$$\begin{array}{c} f\left(\eta \right)=f_{0}\left(\eta \right)+\sum_{m=1}^{\infty }f_{m}\left(\eta \right), \end{array}$$ (28)

$$\begin{array}{c} g\left(\eta \right)=g_{0}\left(\eta \right)+\sum_{m=1}^{\infty }g_{m}\left(\eta \right), \end{array}$$ (29)

$$\begin{array}{c} \theta \left(\eta \right)=\theta_{0}\left(\eta \right)+\sum_{m=1}^{\infty }\theta_{m}\left(\eta \right). \end{array}$$ (30)

The problems at mth-order satisfy the following:

$$\begin{array}{cc} & L_{f}\left[f_{m}\left(\eta \right)-\chi_{m}f_{m-1}\left(\eta \right)\right]=\hbar R_{m}^{f}\left(\eta \right), \end{array}$$ (31)

$$\begin{array}{cc} & L_{g}\left[g_{m}\left(\eta \right)-\chi_{m}g_{m-1}\left(\eta \right)\right]=\hbar R_{m}^{g}\left(\eta \right), \end{array}$$ (32)

$$\begin{array}{cc} & L_{\theta }\left[\theta_{m}\left(\eta \right)-\chi_{m}\theta_{m-1}\left(\eta \right)\right]=\hbar R_{m}^{\theta }\left(\eta \right), \end{array}$$ (33)

where

$$\begin{array}{cc} & f_{m}\left(0\right)=f_{m}^{\prime }\left(0\right)=g_{m}\left(0\right)=g_{m}^{\prime }\left(0\right)=\theta_{m}\left(0\right)=0,\\ & f_{m}^{\prime }\left(\infty \right)=g_{m}^{\prime }\left(\infty \right)=\theta_{m}\left(\infty \right)=0. \end{array}$$ (34)

Here

$$\begin{array}{c} R_{m}^{f}\left(\eta \right)=f_{m-1}^{\prime \prime \prime }+\left(M^{2}\beta +1\right)\sum_{k=0}^{m-1}\left[\left(f_{m-1-k}+g_{m-1-k}\right)f_{k}^{\prime \prime }-f_{m-1-k}^{\prime }f_{k}^{\prime }\right]+\beta \sum_{k=0}^{m-1}[2\\ \left(f_{m-1-k}+g_{m-1-k}\right)\sum_{l=0}^{k}f_{k-l}^{\prime }f_{l}^{\prime \prime }-(f_{m-1-k}\sum_{l=0}^{k}f_{k-l}+g_{m-1-k}\sum_{l=0}^{k}g_{k-l}+\\ 2f_{m-1-k}\sum_{l=0}^{k}g_{k-l})f_{l}^{\prime \prime \prime }]-M^{2}f_{m-1}^{\prime }, \end{array}$$ (35)

$$\begin{array}{c} R_{m}^{g}\left(\eta \right)=g_{m-1}^{\prime \prime \prime }+\left(M^{2}\beta +1\right)\sum_{k=0}^{m-1}\left[\left(f_{m-1-k}+g_{m-1-k}\right)g_{k}^{\prime \prime }-g_{m-1-k}^{\prime }g_{k}^{\prime }\right]+\beta \sum_{k=0}^{m-1}[2\\ \left(f_{m-1-k}+g_{m-1-k}\right)\sum_{l=0}^{k}g_{k-l}^{\prime }g_{l}^{\prime \prime }-(f_{m-1-k}\sum_{l=0}^{k}f_{k-l}+g_{m-1-k}\sum_{l=0}^{k}g_{k-l}+2f_{m-1-k}\\ \sum_{l=0}^{k}g_{k-l})g_{l}^{\prime \prime \prime }]-M^{2}g_{m-1}^{\prime }, \end{array}$$ (36)

$$\begin{array}{c} R_{m}^{\theta }\left(\eta \right)=\frac{1}{Pr}\theta_{m-1}^{\prime \prime }+\sum_{k=0}^{m-1}\left[\left(f_{m-1-k}+g_{m-1-k}\right)\theta_{k}^{\prime }\right]-\gamma \sum_{k=0}^{m-1}[2\left(f_{m-1-k}+g_{m-1-k}\right)\\ \sum_{l=0}^{k}\left(f_{k-l}^{\prime }+g_{k-l}^{\prime }\right)\theta_{l}^{\prime }+(f_{m-1-k}\sum_{l=0}^{k}f_{k-l}+g_{m-1-k}\sum_{l=0}^{k}g_{k-l}+2f_{m-1-k}\\ \sum_{l=0}^{k}g_{k-l})\theta_{l}^{\prime \prime }], \end{array}$$ (37)

$$\chi_{m}=\{\begin{array}{cc} 0, & \text{if }m\leq \text{1}\\ 1, & \text{if }m>\text{1} \end{array}.$$

Eqs (31)–(34) are linear and can be solved exactly by using computational software MATHEMATICA for different values of *m*. When *β* = *γ* = 0.25, *Pr* = 1, *M* = λ = 0.5 and ℏ = −0.8, the solutions containing first four terms are as under:

$$\begin{array}{cc} f(\eta )= & \frac{1}{19353600000}e^{-7\eta }(603525-5921786e^{\eta }+13057328914e^{7\eta }-7e^{2\eta }(2091179+2045250\\ & \eta )+14e^{3\eta }\left(29205533+5324400\eta \right)+35e^{4\eta }\left(-34260887+34637760\eta +4920750\eta^{2}\right)\\ & +210e^{5\eta }\left(13036859-19327940+7533000e^{2}\right)+3e^{6\eta }(-4994953069+3521387310\eta \\ & -1243100250\eta^{2}+191362500\eta^{3})). \end{array}$$

$$\begin{array}{cc} g(\eta )= & e^{-28\eta }(-0.00145444e^{21\eta }+0.0289861e^{22\eta }+e^{24\eta }\left(0.733927-0.217469\eta \right)+e^{23\eta }(-0.211133\\ & +0.0152893\eta )+e^{25\eta }\left(-1.32775+0.929162\eta -0.0400452\eta^{2}\right)+e^{26\eta }(1.51166-1.43126\eta \\ & +0.311133\eta^{2})+e^{27\eta }\left(-1.30892+0.8552\eta -0.200826\eta^{2}+0.0148315\eta^{3}\right)). \end{array}$$

$$\begin{array}{cc} \theta (\eta )= & e^{-28\eta }(-0.0488963e^{21\eta }+0.595323e^{22\eta }+e^{24\eta }\left(6.32871-2.32886\eta \right)+e^{23\eta }(-2.76871+\\ & 0.33542\eta )+e^{25\eta }\left(-7.85534+5.18209\eta -0.405422\eta^{2}\right)+e^{26\eta }(5.70957-4.48284\eta \\ & ++0.828883\eta^{2})e^{27\eta }\left(-2.68653+1.1697\eta -0.208538\eta^{2}+0.0127943\eta^{3}\right)). \end{array}$$

## Convergence of homotopy series solutions

**Fig 2:**
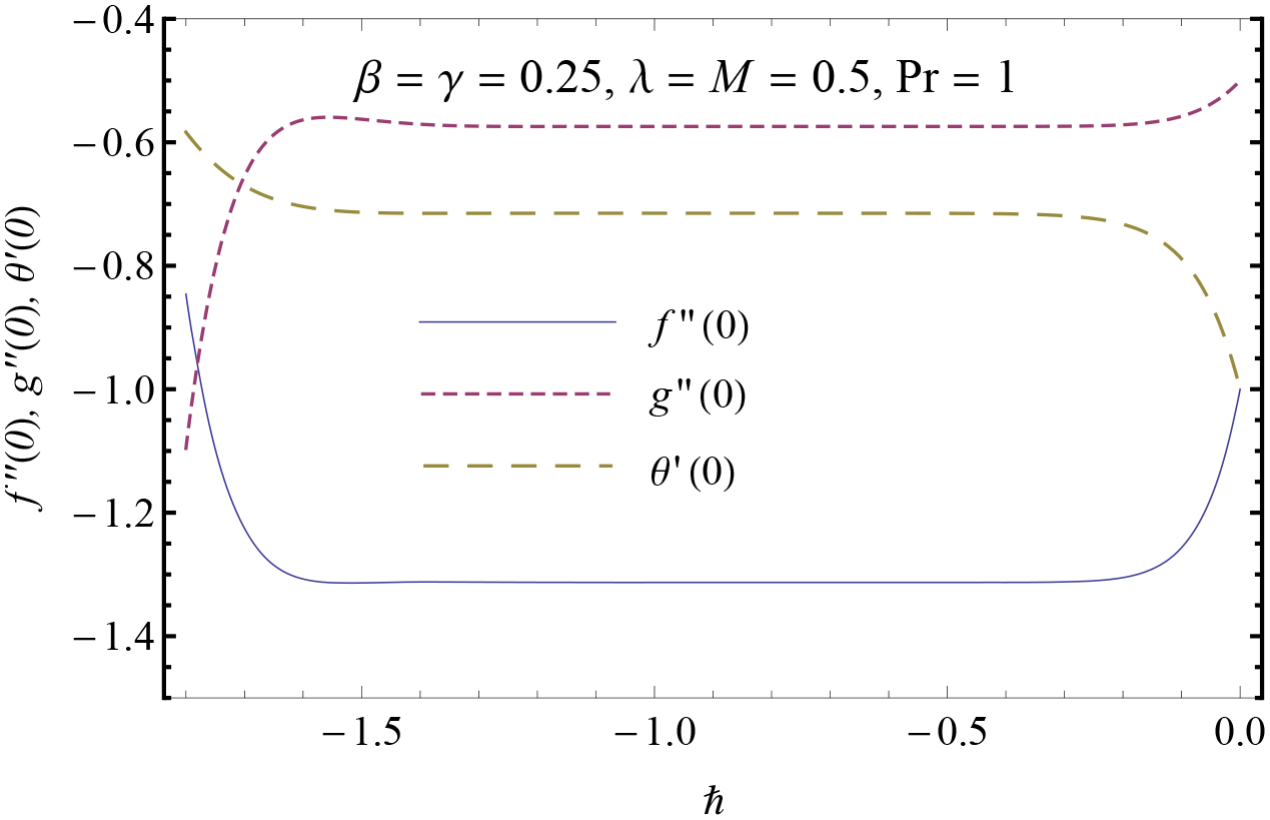
ℏ− curves for the functions *f*(*η*), *g*(*η*) and *θ*(*η*).

Note that the series solutions given in Eqs (28)–(30) contain an auxiliary parameters ℏ which has an important role in controlling the convergence of homotopic solutions. To select an appropriate value of ℏ, we have plotted the so-called ℏ− curves for *f*′′(0), *g*′′(0) and *θ*′(0) in Fig 2. Here the valid range of ℏ lies where the ℏ− curves are parallel to ℏ− axis. From Fig 2, we expect that series solutions for *f*, *g* and *θ* would converge in the range −1.5 ≤ ℏ ≤ −0.4. Table 1 is plotted to see the convergence rate of the solutions. We observe that tenth-order approximations are sufficient for convergent solutions at ℏ = −0.8.

**Table 1:** Convergence of HAM solutions for different orders of approximations when *β* = *γ* = 0.25, *Pr* = 1, *M* = λ = 0.5 and ℏ = −0.8.

*Order of approximations*	*f*′′(0)	*g*′′(0)	*θ*′(0)
5	-1.31282	-0.57423	-0.71696
10	-1.31296	-0.57435	-0.71497
15	-1.31296	-0.57435	-0.71492
20	-1.31296	-0.57435	-0.71491
25	-1.31296	-0.57435	-0.71491
30	-1.31296	-0.57435	-0.71491
35	-1.31296	-0.57435	-0.71491
40	-1.31296	-0.57435	-0.71491

## Results and Discussion

**Fig 3:**
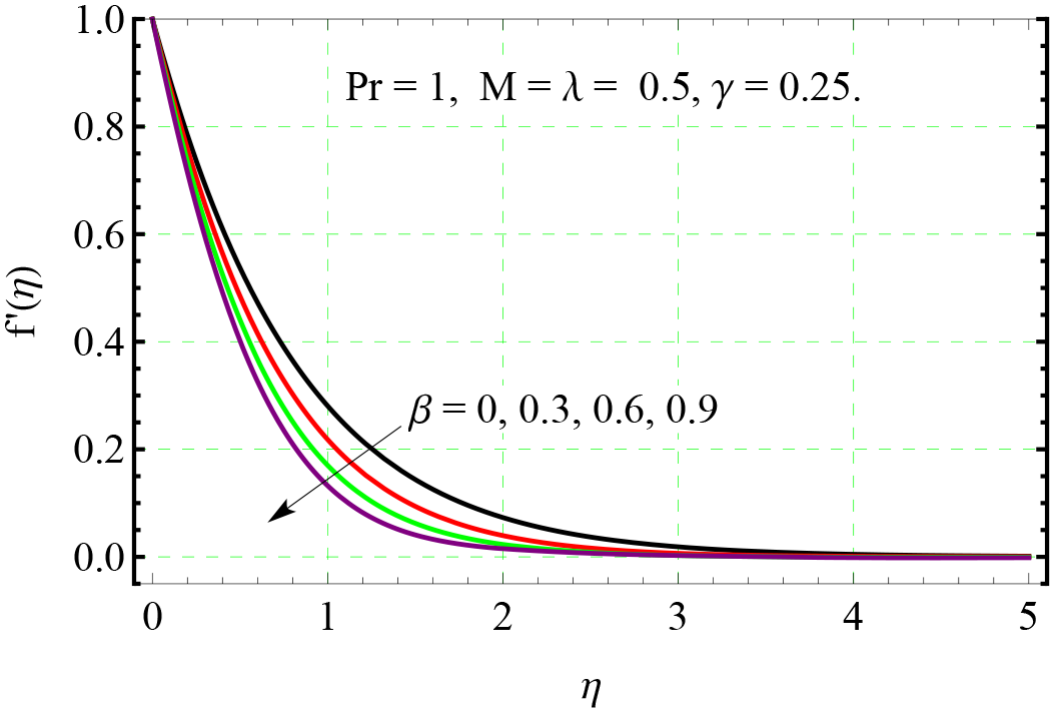
Effect of *β* on *f*′(*η*).

**Fig 4:**
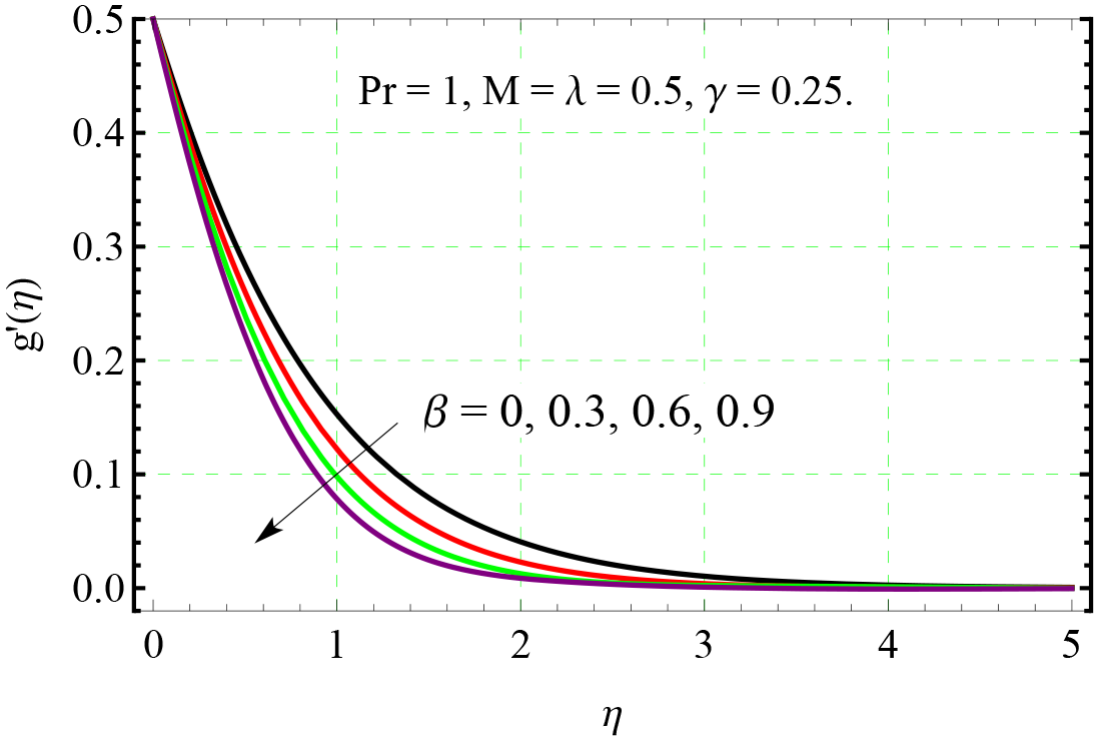
Effect of *β* on *g*′(*η*).

**Fig 5:**
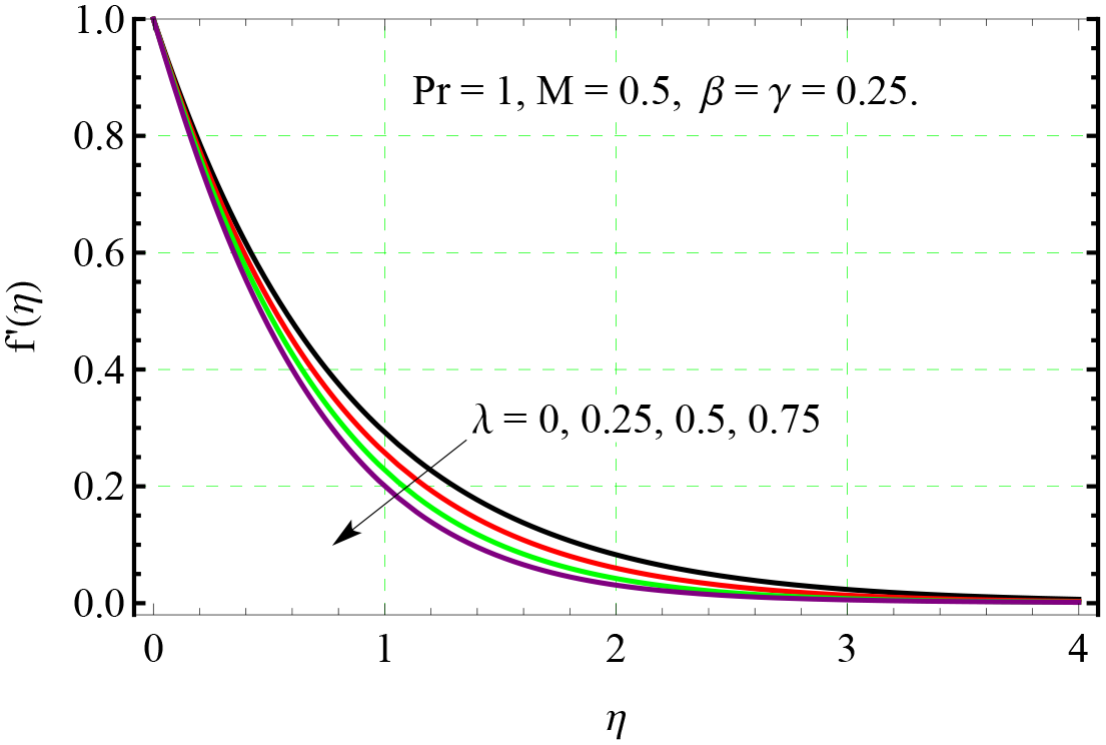
Effect of λ on *f*′(*η*).

**Fig 6:**
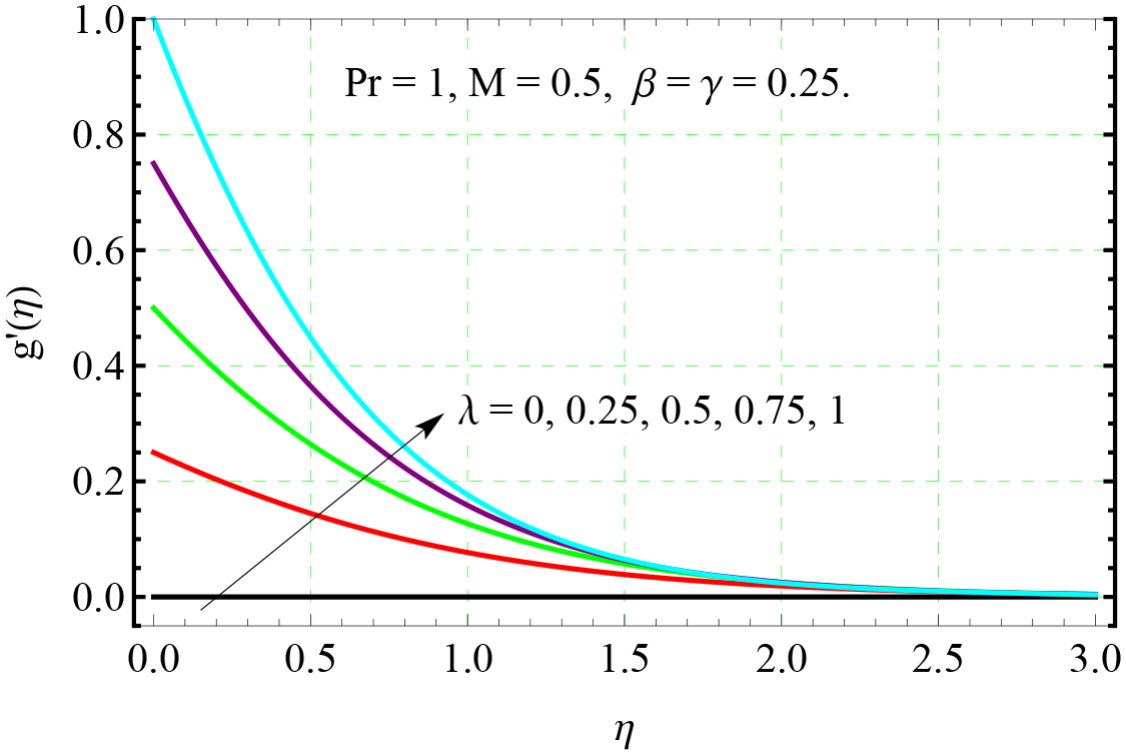
Effect of λ on *g*′(*η*).

**Fig 7:**
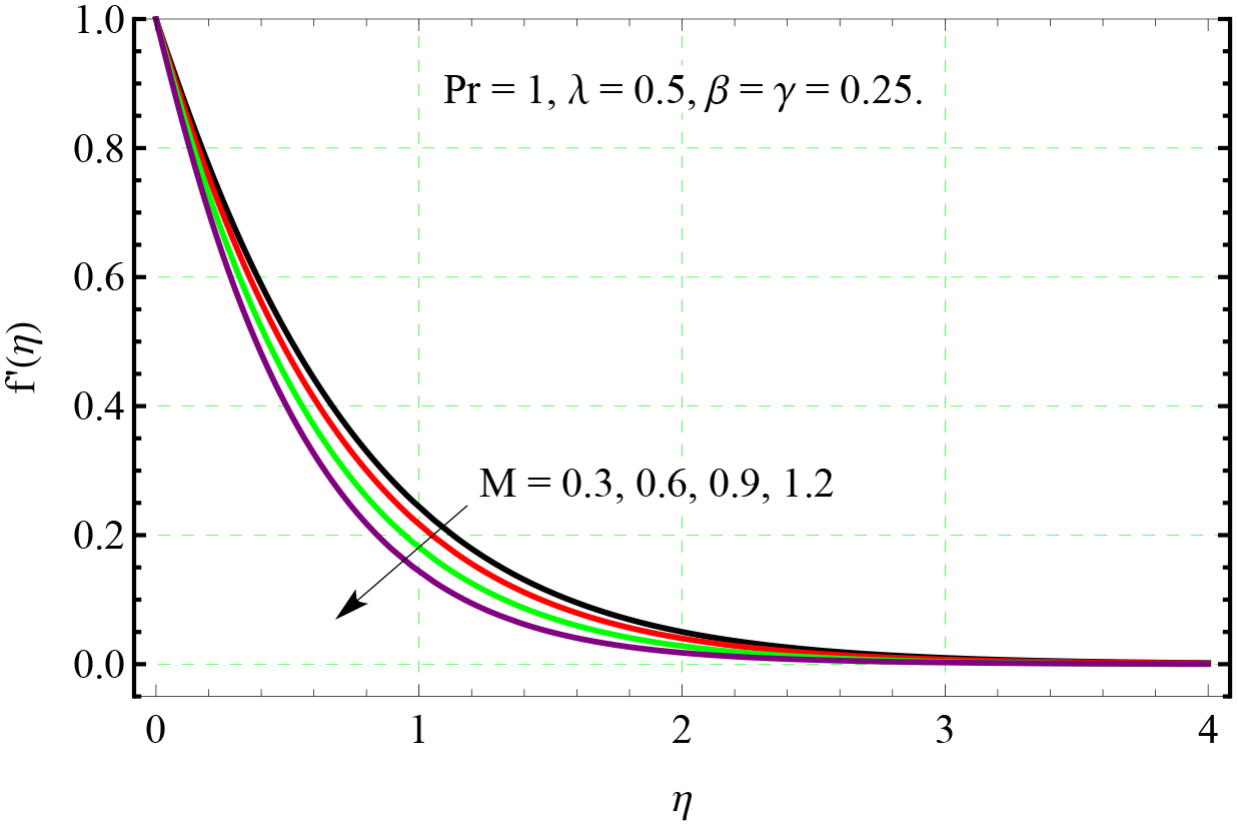
Effect of *M* on *f*′(*η*).

**Fig 8:**
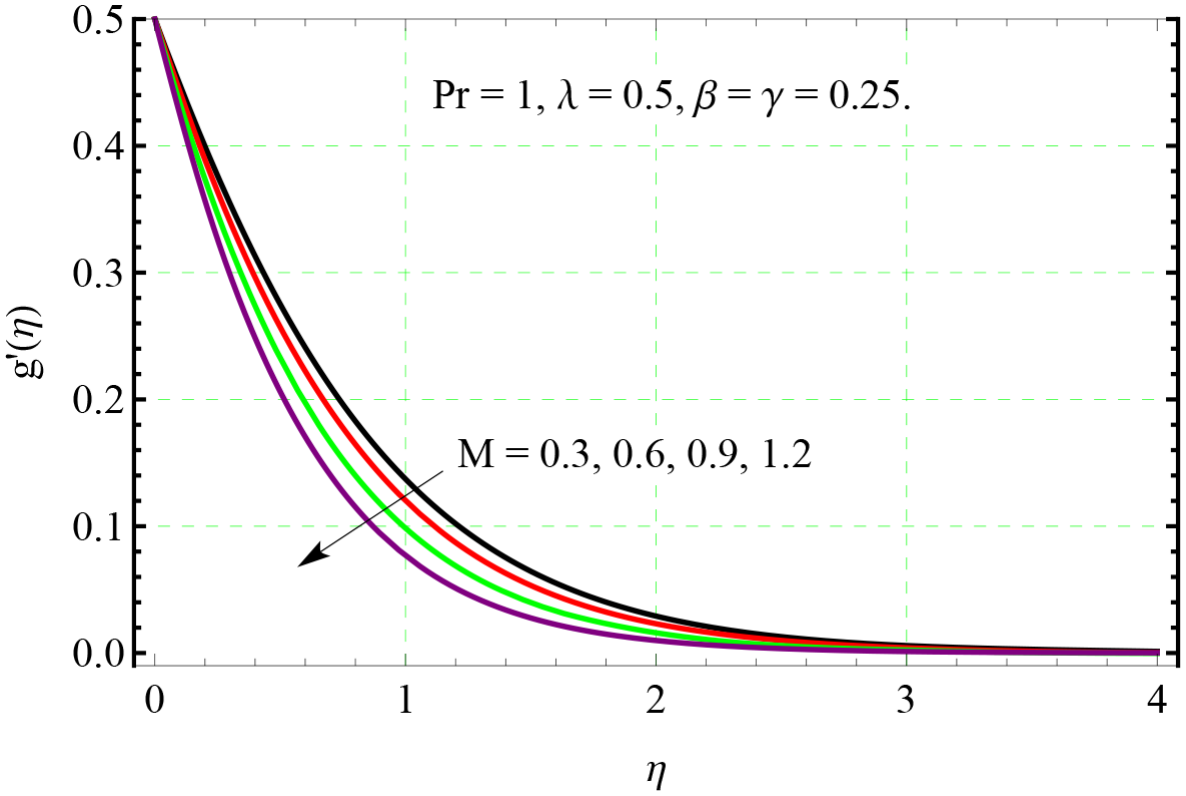
Effect of *M* on *g*′(*η*).

**Fig 9:**
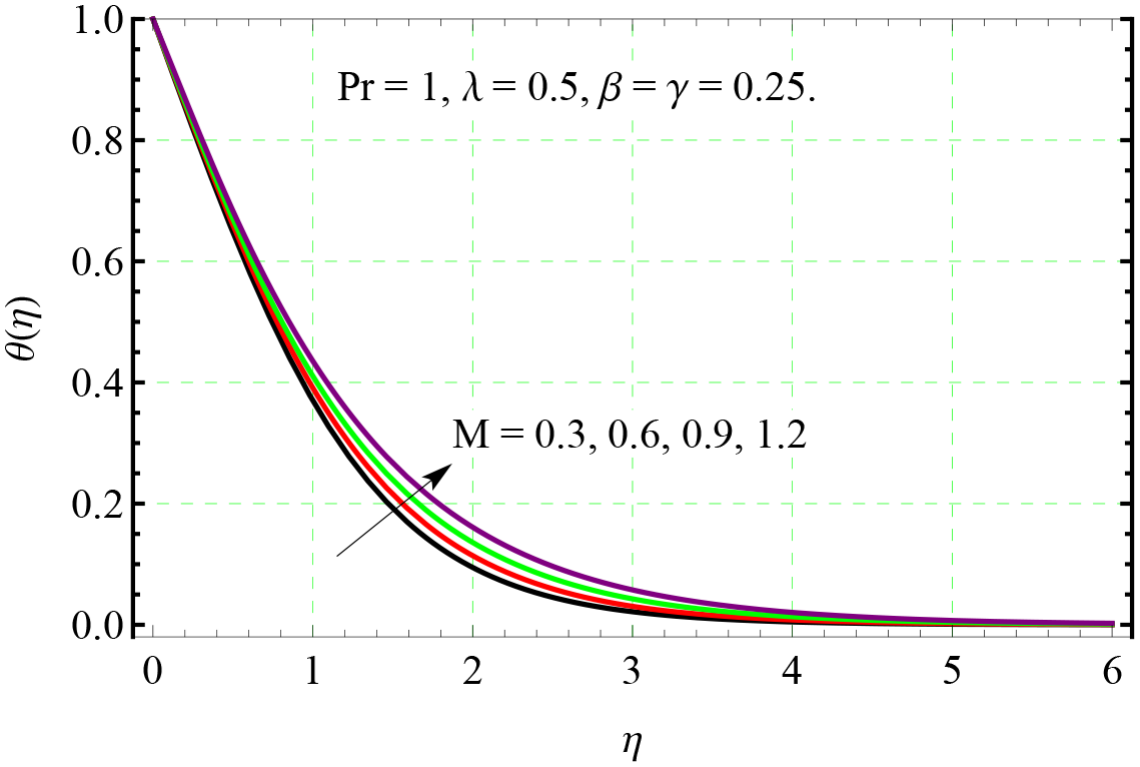
Effect of *M* on *θ*(*η*).

**Fig 10:**
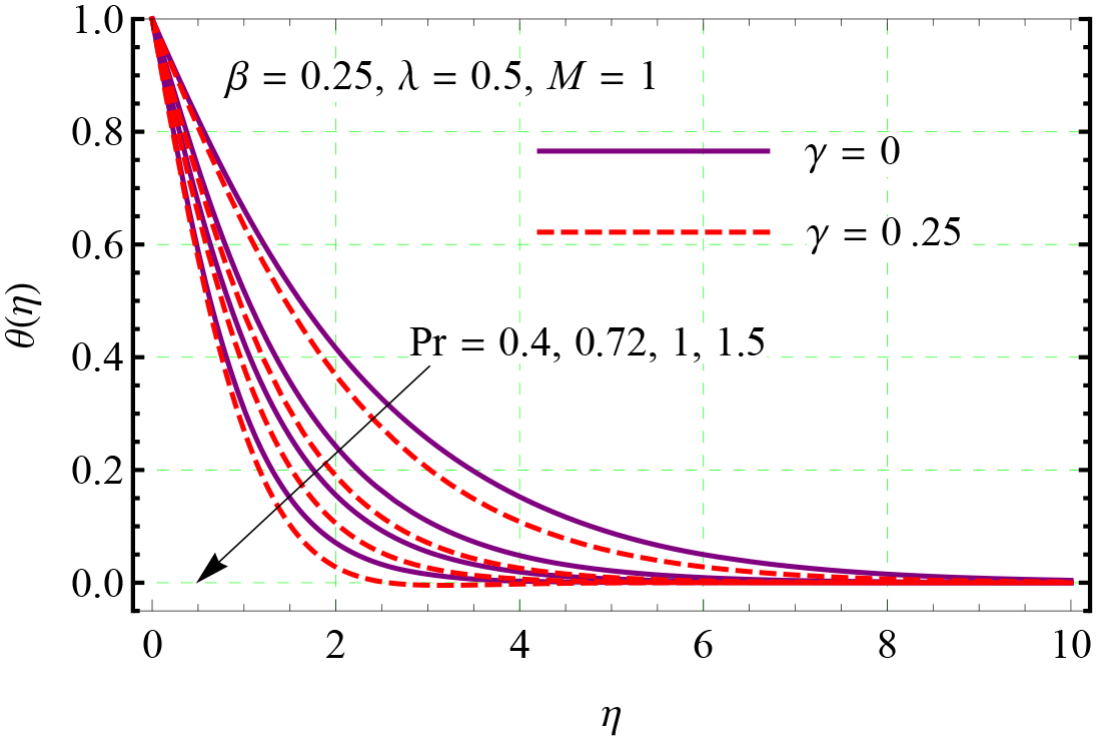
Effect of *Pr* and *γ* on *θ*(*η*).

**Fig 11:**
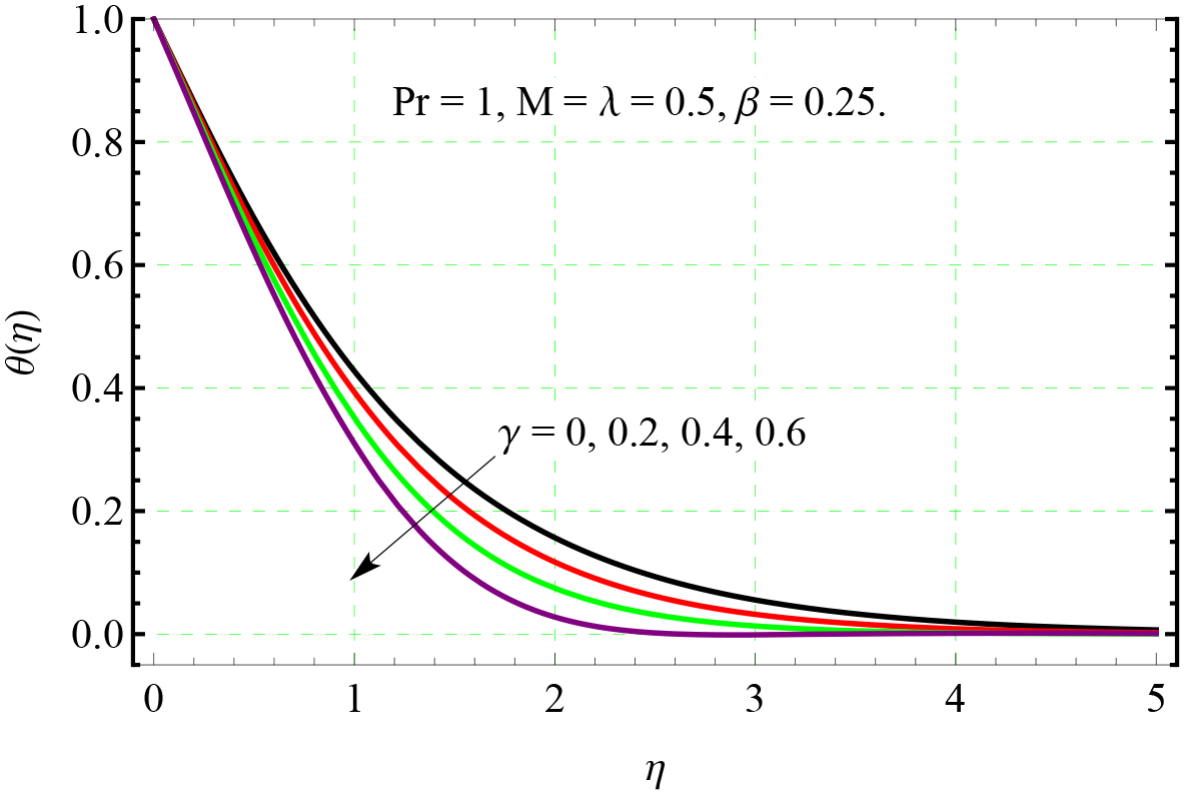
Effect of *γ* on *θ*(*η*).

**Fig 12:**
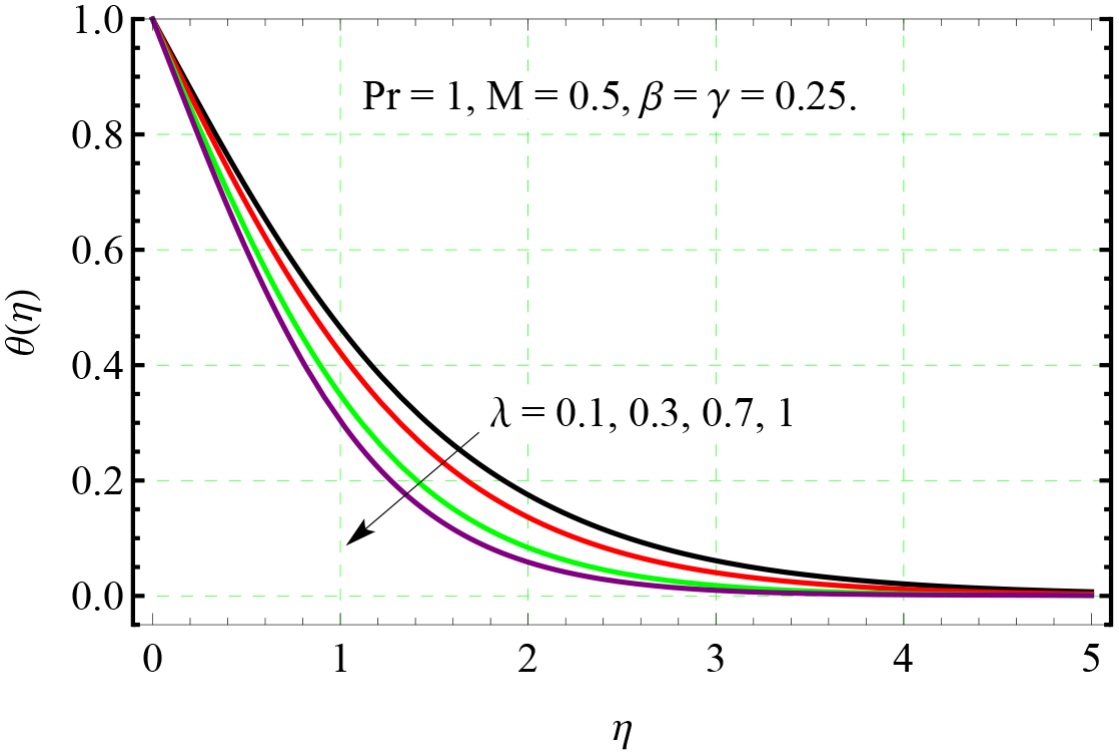
Effect of λ on *θ*(*η*).

This section focuses on the physical interpretation of the behaviour of the embedded parameters on the solutions. For this purpose, we display graphical results in Figs 3–12. Table 2 includes the numerical values of wall temperature gradient *θ*′(0) for different value of *β*, *γ* and *M*. The entries of this table are obtained at suitable choice of ℏ. It is observed that *θ*′(0) has direct relationship with the thermal relaxation time. However it is a decreasing function of the fluid relaxation time *β*. The presence of magnetic field also causes diminution in the magnitude of heat transfer rate from the surface.

**Table 2:** Values of wall temperature gradient *θ*′(0) for different value of *β*, *γ*, *M* when ℏ = −0.8 *Pr* = 1 and λ = 0.5.

*β*	*γ*	*M*	*θ*′(0)
0	0.25	0.5	-0.75689
0.2			-0.72298
0.4			-0.69171
0.6			-0.66313
0.25	0		-0.67657
	0.2		-0.70680
	0.4		-0.74072
	0.6		-0.77877
	0.25	0	-0.74203
		0.5	-0.71491
		1	-0.64859

The behavior of non-dimensional relaxation time *β* on both the *x*− and *y*− components of velocity can be observed from Figs 3 and 4 respectively. The velocity profiles are tilted towards the wall when *β* is increased indicating that velocity and boundary layer thickness are decreasing function of *β*.

Physically, bigger *β* indicates stronger viscous force which restricts the fluid motion and consequently the velocity decreases. Figs 5 and 6 show the impact of stretching rates ratio λ on the velocity fields *f*′ and *g*′ respectively. Bigger values of λ indicates larger rate of stretching along the *y*− direction compared to *x*− direction. Therefore, with an increase in λ, the velocity in the *y*− direction increases and velocity in the original *x*− direction decreases simultaneously.

In Figs 7 and 8, the velocity distributions are presented for different value of Hartman number *M*. Velocities in both *x*− and *y*− directions decrease upon increasing the *M*. This decrease in the velocity is due to resistance offered by the Lorentz force acting in the normal direction. From Fig 9, we observe that the resistance associated with Lorentz force supports the penetration depth of temperature.

In Fig 10, the temperature profiles are presented for different Prandtl numbers. Here *γ* = 0 indicates the corresponding results for the classical Fourier law. Prandtl number has inverse relationship with thermal diffusivity. Therefore an increase in Pr reduces conduction and hence causes a reduction in the penetration depth of temperature. The results are qualitatively similar in both Fourier and Cattaneo-Christov heat flux models.

The effects of non-dimensional relaxation time *γ* on temperature distribution can be analyzed from Fig 11. Temperature *θ* decreases and profiles smoothly descend to zero at shorter distance from the sheet when *γ* is incremented. This indicates that there will be thinner thermal boundary layer when relaxation time for heat flux is larger. Here the profiles become steeper in the vicinity of the boundary as *γ* increases which is an indication of the growth in wall slope of temperature *θ*.

The impact of stretching rates ratio on the temperature distribution can be analyzed through Fig 12. Although we do not include the results for entrainment velocity here but our computations indicate that entrainment velocity *f*(∞) + *g*(∞) is an increasing function of λ. Due to this reason, an increase in λ enhances the intensity of the cold fluid at the ambient towards the hot stretching surface. Consequently the fluid temperature drops within the boundary layer, when λ is increased.

## Conclusions

Cattaneo-Christov heat flux model is employed to study the MHD three-dimensional viscoelastic flow above a bi-directional stretching surface. The problems are first modeled and then solved via HAM for different values of the parameters. The main results of this work are listed below:

- The velocity and boundary layer thickness are decreasing functions of the fluid relaxation time λ_1_.
- The velocity gradients *f*′′(0) and *g*′′(0) are found to increase upon increasing the fluid relaxation time λ_1_.
- Hartman number *M* supports the thickness of thermal boundary layer.
- Temperature and thermal boundary layer have inverse relationship with relaxation time for heat flux λ_2_.
- The vertical component of velocity at far field boundary increases when stretching rates ratio λ is increased.
- The behavior of fluid relaxation time λ_1_ is qualitatively similar in both Fourier and Cattaneo-Christov heat flux models.
- The present model reduces to the case of Newtonian fluid by choosing *β* = 0.
